# Reliability tests and validation tests of the client satisfaction questionnaire (CSQ-8) as an index of satisfaction with childbirth-related care among Filipino women

**DOI:** 10.1186/1471-2393-13-235

**Published:** 2013-12-17

**Authors:** Chieko Matsubara, Joseph Green, Linda Teresa Astorga, Edgardo L Daya, Honorato C Jervoso, Edgardo M Gonzaga, Masamine Jimba

**Affiliations:** 1 Graduate School of Medicine, The University of Tokyo, 7-3-1, Hongo, Bunkyo-ku, Tokyo 113-0033, Japan; 2 Provincial Health Office, Leyte Province, Philippines; 3 Western Leyte Provincial Hospital, Baybay, Leyte, Philippines; 4 Department of Health - Center for Health Development, Southern Tagalog (Calabarzon-A), Calamba, Philippines; 5 Bureau of International Medical Cooperation, National Center for Global Health and Medicine, 1-21-1, Toyama, Shinjuku-ku, Tokyo 162-8655, Japan

## Abstract

**Background:**

Satisfaction is an important indicator of the quality of care during childbirth. Previous research found that a good environment at a health facility can increase the number of deliveries at that facility. In contrast, an unsatisfying childbirth experience could cause postpartum mental disorder. Therefore it is important to measure mothers’ satisfaction with their childbirth experiences. We tested whether the eight-item Client Satisfaction Questionnaire (CSQ-8) provided useful information about satisfaction with childbirth-related care. The government of the Philippines promotes childbirth at health facilities, so we tested the CSQ-8 in the Philippine cities of Ormoc and Palo.

**Methods:**

This was a cross-sectional study. We targeted multigravid mothers whose last baby had been delivered at a hospital (without complications) and whose 2nd-to-last baby had been delivered at a hospital or at home (without complications). We developed versions of the CSQ-8 in Cebuano and Waray, which are two of the six major Filipino languages. Reliability tests and validation tests were done with data from 100 Cebuano-speaking mothers and 106 Waray-speaking mothers.

**Results:**

Both the Cebuano and Waray versions of the CSQ-8 had high coefficients of internal-consistency reliability (greater than 0.80). Both versions were also unidimensional, which is generally consistent with the English CSQ-8 in a mental-health setting. As hypothesized, the scores for data regarding the second-to-last delivery were higher for mothers who had both their second-to-last and their last delivery in a hospital, than for mothers who had their second-to-last delivery at home and their last delivery in a hospital (Cebuano: p < 0.001, rho = 0.51, Waray: p < 0.001, rho = 0.55).

**Conclusions:**

Scores on the CSQ-8 can be used as indices of general satisfaction with childbirth-related services in clinical settings. This study also exemplifies a convenient method for developing versions of the CSQ-8 in more than one language. These versions of the CSQ-8 can now be used to assess mothers’ satisfaction, so that mothers’ opinions can be taken into account in efforts to improve childbirth-related services, which could increase the proportion of deliveries in medical facilities and thus reduce maternal mortality.

## Background

Satisfaction is an important indicator of the quality of care during childbirth [1-4]. For example, in Brazil, after healthcare managers improved delivery rooms, pregnant women were more satisfied and the number of deliveries at that health facility increased [5]. To provide better birth environments, healthcare managers and policy makers have tried to understand mothers’ experiences [6-8]. If a birth enviroment is not satisfactory, childbirth experiences could cause postpartum mental problems [9,10]. Hence, creating a satisfactory environment for childbirth is imperative.

High maternal satisfaction is an important concern in the Philippines. To achieve Millenium Development Goal 5, the Philippines has committed itself to reducing its maternal mortality ratio (MMR): from 180 to 45 per 100,000 live births by 2015 [11]. The MMR in the Philippines sharply declined initially, but it was still 94 per 100,000 in 2008 [11,12]. The high MMR in the Philippines was attributed to the predominance of home births (56% in 2008) and the high proportion of those births that were assisted by traditional birth attendants (36% in 2008) [13]. Access to health services for the poor was hampered by economic, physical, and socio-cultural barriers [14]. Furthermore, some Filipinos are hesitant to go to health facilities even if they are conveniently located [15,16] because some health professionals treat poor people inappropriately [17]. Assessing maternal satisfaction is thus important to promote childbirth in medical facilities.

Questionnaires that might be used to measure maternal satisfaction are available [1-4,18]. However, those scales are unwieldy to use in clinical settings and they require cultural adaptation. Furthermore, the dimensions comprising satisfaction differ among countries: Tangibles and empathy may be more important in Asia, while responsiveness, reliability, and assurance may be more important in the West [19]. Partner participation is important in the United States [4] but not in Turkey [18].

Such differences make it difficult to use one scale in more than one country. Two studies [1,3] included global satisfaction scales, but they measured satisfaction with childbirth itself and did not assess the determinants of satisfaction with care comprising global satisfaction. In one review, patients’ demographics were found to be the most important determinants of general satisfaction with healthcare, while satisfaction with childbirth experiences had little or no relationship with demographics [20]. We need a scale to measure general satisfaction with childbirth-related care, and we need information from reliability testing and validation testing of that scale.

The Client Satisfaction Questionnaire (CSQ-8) might meet those needs. The CSQ-8 was developed to assess global client satisfaction, along a single dimension, in a clinical setting in the United States [21]. The CSQ-8 has eight question-items (quality of service, kind of service, met needs, recommend to a friend, amount of help, deal with problems, overall satisfaction, and come back; see Additional file 1). Clients respond to those question-items using a 4-point Likert scale. Their responses are scored from 1 to 4, and thus the possible total scores range from 8 to 32. Higher scores indicate greater satisfaction. Reliability testing and validation testing of the CSQ-8 have been done in mental-health clinical contexts [21-23], and the CSQ-8 has been translated into more than 20 languages [24].

The CSQ-8 has been used at least once before to measure satisfaction with childbirth care. In Japan, a modified Japanese-language mental-health CSQ-8 was used in the context of childbirth care, but results of reliability tests and validation tests were not reported [25]. Although this scale might be useful for measuring satisfaction with childbirth care, reliability and validity in specific situations should not be assumed, and therefore they should be tested.

We developed versions of the CSQ-8 in two Filipino languages (Cebuano and Waray), and here we report the results of reliability testing and validation testing of those two questionnaires, with the aim of evaluating whether the CSQ-8 could be used to assess satisfaction with childbirth care in the Philippines.

## Methods

### Translation of the CSQ-8

Many languages are spoken in the Philippines. The six major languages are Tagalog, Cebuano, Ilocano, Bicol, Hiligaynon, and Waray [13]. For this study we selected Cebuano and Waray. Those two languages are the predominant languages in Biliran Province, which had the worst MMR in 2005 in the Philippines [26]. In 2000, 13.1% of Filipinos spoke Cebuano and 10.9% spoke Waray [27].

We modified the CSQ-8 to fit the context of childbirth (Additional file 1). An expert in the health sector, who is a native Cebuano speaker, translated the modified CSQ-8 from English into Cebuano. Eight registered nurses then translated the Cebuano version back into English, and they revised the Cebuano CSQ-8 to improve translation accuracy. Using the same procedure, another expert and eight other registered nurses prepared the Waray CSQ-8.

As Waray has no words equivalent in meaning to the English word “care”, we used “service” instead. (Cebuano has words equivalent to “care” and to “service”). Three provincial health officers, who were native speakers of both Cebuano and Waray, independently reviewed the Cebuano CSQ-8 and the Waray CSQ-8 and verified that the translations were appropriate and that there were no differences between the Cebuano and Waray versions in the meaning of each item.

### Study site

This was a cross-sectional study. The settings were Leyte Provincial Hospital in Palo city and Ormoc District Hospital in Ormoc city. Cebuano is the main language spoken in Ormoc city and Waray is spoken in Palo city.

We selected these two hospitals for the following three reasons. First, both of them are in the semi-urban areas of Tacloban city and Ormoc city in Leyte Province. Second, they are public hospitals and are ranked as referral hospitals (Level 2). They receive patients from first-level health facilities. Third, both of them are leading hospitals in a maternal and child health project that the Department of Health of the Philippines and the Japan International Cooperation Agency (JICA) launched in Region VIII in 2010 (Phase II) [28].

### Target population for testing the CSQ-8

We targeted multigravid mothers whose last baby had been delivered at a hospital, without complications, and whose 2nd-to-last baby had been delivered either at a hospital or at home without complications. The participants were recruited in the post-delivery rooms of the two hospitals. To enlist participants, research assistants (interviewers) visited the post-delivery rooms and asked mothers individually. We included mothers who had delivered the last baby at hospital, were multigravid, and had delivered without complications at both their last and 2nd-to-last pregnancies and deliveries. If the mother met those criteria, then the interviewers explained the study and invited her to participate. Ensuring a person-to-item ratio of at least 10:1 [29] for the eight CSQ-8 items, the total number of participants was 100 for the Cebuano CSQ-8 and 106 for the Waray CSQ-8.

### Preliminary testing of the CSQ-8

Before the main survey, we tested the CSQ-8 with 100 mothers (50 at Ormoc and 50 at Palo) to determine if all items were understandable, if each question had appropriate response choices, and if the participants were comfortable completing the survey. Translators partially revised some items in the CSQ-8 to make the expressions more appropriate and usable in the whole study area. This is because the expressions were different from municipality to municipality even in the same language (especially in Waray). Also, the interviewers found that the participants were not familiar with the use of the Likert-type scale.

### Independent variables for testing the CSQ-8

Participants at the Ormoc District Hospital were asked about the place of the previous delivery and about demographics. Participants at Leyte Provincial Hospital were also asked about the place of the previous delivery, and their demographic information was obtained from medical records after the interviews.

### Reliability testing and validation testing

We used Cronbach’s alpha as the index of internal-consistency reliability. Provincial health officers assessed content validity [30]. In its original English-language version and in the context in which it was originally used, the CSQ-8 was found to be one-dimensional. To find out whether that was also true of the Cebuano and Waray CSQ-8, we conducted exploratory principal-components analysis.

For construct-validation testing, we tested the following hypothesis, which is based on the relationship between utilization and satisfaction with reproductive healthcare that was found in a study done in Vietnam [31]: “Higher levels of client satisfaction are positively related to future clinic use intentions”.

As this study was cross-sectional, we could not test predictions about future behaviors. Instead, we reasoned that mothers who were satisfied with the care they had received in a hospital when their 2^nd^-to-last baby was delivered would return to a hospital for the next delivery, and that mothers who were *not* satisfied with the care they had received *at home* when their 2^nd^-to-last baby was delivered would go to a hospital for the next delivery. The former were mothers whose 2^nd^-to-last baby had been born in a hospital, and the latter were mothers whose 2^nd^-to-last baby had been born at home. Therefore we tested the following hypothesis: “Mothers who successively delivered their babies at hospitals (without complications) had higher satisfaction with their 2^nd^-to-last deliveries compared with those who had delivered their 2^nd^-to-last babies at home (without complications) and delivered their last baby at hospitals (without complications).” As with all validation studies, the results of this hypothesis test indicate whether the instrument (in this case, the CSQ-8) actually measures what it is intended to measure. If the results are consistent with the hypothesis, then we can reasonably conclude that the CSQ-8 does measure satisfaction of these women with the childbirth services they received. In that case, healthcare providers and policymakers can have confidence that CSQ-8 scores in fact are an index of satisfaction with childbirth services.

We compared the satisfaction scores for 2^nd^-to-last deliveries by the place of delivery (hospital vs. home). This required the mothers to recall their satisfaction with the 2^nd^-to-last delivery, and in this context we note that in two previous studies mothers’ satisfaction ratings in the immediate post-partum period were very similar to those even 15 to 20 years later [32,33].

### Data collection for testing the CSQ-8

We collected data from May to August, 2011. After undergoing one day of training, eight interviewers collected data in the post-delivery rooms of the two hospitals. The interviewers completed the questionnaire based on the participants’ responses. To ensure that the participants distinguished as clearly as possible between their last childbirth experience and their 2nd-to-last childbirth experience, the interviewers first recorded the names of the last and 2nd-to-last babies.

### Data analysis for testing the CSQ-8

SPSS version 12.1 for Windows was used to analyze the data. As the CSQ-8 scores were not normally distributed, we used Spearman’s rank-order correlation coefficient to analyze the correlations of CSQ-8 scores with age and with the number of previous deliveries. For the correlations of CSQ-8 scores with health insurance coverage and with occupation, we used the point-biserial correlation. As mentioned above, we also performed principal-components analysis. Before the principal-components analysis, we checked the suitability of the data for that analysis by the Kaiser-Meyer-Olkin values and Bartlett’s Test of Sphericity. We used the Mann–Whitney U test to compare the CSQ-8 scores for the 2nd-to-last delivery between home and hospital.

### Ethical considerations

The Research Ethics Committee of the University of Tokyo’s Graduate School of Medicine and the Regional Health Research Development Consortium in Region VIII, the Philippines, examined and approved the plan for this study. Following the approvals, the directors of Leyte Provincial Hospital and Ormoc District Hospital examined and approved the study. Mothers’ participation was voluntary, and we kept all data confidential. Before collecting data, we informed the participants of the aim of the study. It was possible for the participants to cease their involvement, as the interviewers had informed the participants of the aim of the study and explained that they could choose to no longer participate. Interviewers then started to interview the respondents who had given their written informed consent. None of the mothers who met the inclusion criteria refused to participate.

## Results

**Figure 1:**
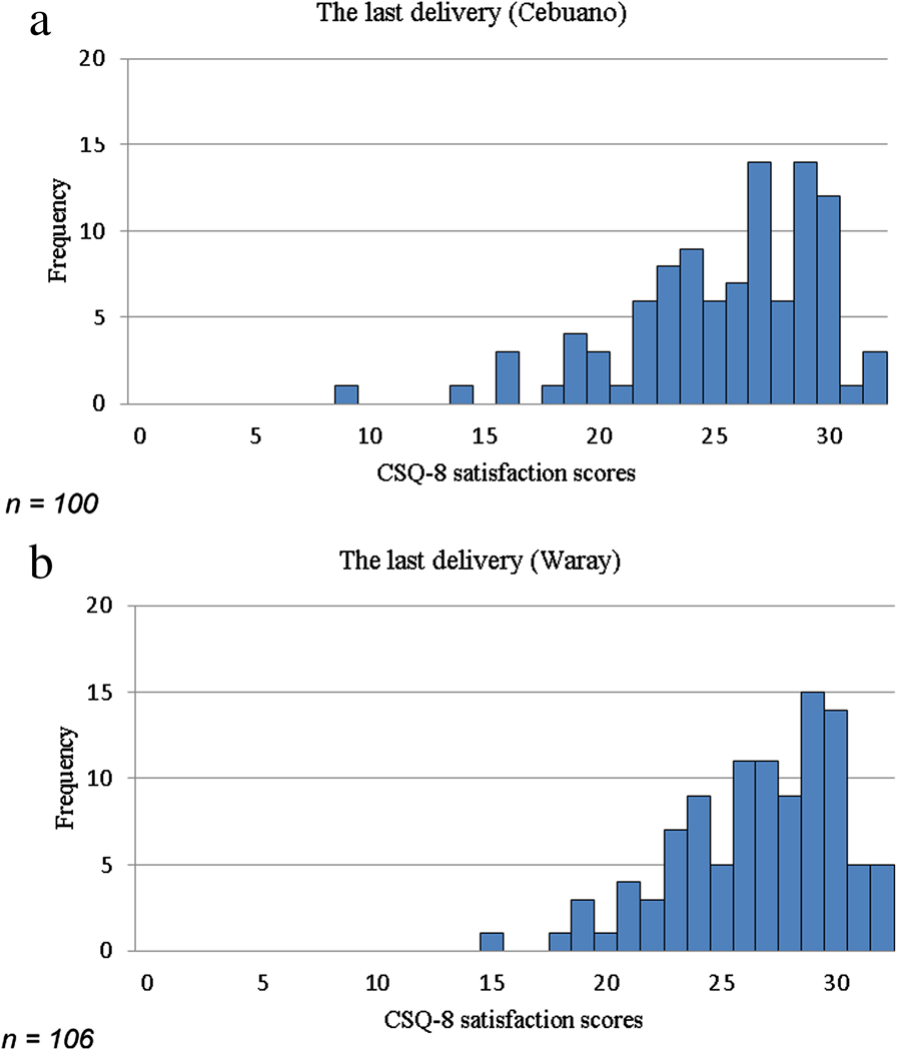
Distribution of CSQ-8 scores for the last delivery, by language (1-a: Cebuano, 1-b: Waray).

Figure 1 shows the CSQ-8 scores of the last deliveries for the Cebuano and Waray versions. On the Cebuano CSQ-8, 25% of the participants had a score greater than 29, and 3.0% had the maximum score of 32 (median = 26.5, 25% to 75% = 29 to 23). On the Waray CSQ-8, 25% of the participants had a score greater than 29, and 4.5% had the maximum score of 32 (median = 27.0, 25% to 75% = 29 to 24).

Table 1 shows the participants’ demographic characteristics and correlations between CSQ-8 scores of their last delivery and demographic characteristics. For Waray-speaking participants, we could not find the characteristics of all participants in the medical records, because not all of the family names mentioned during the interview matched those written in the medical record (maiden name or married name). Statistically significant associations were not detected between CSQ-8 scores and age, number of previous deliveries, health insurance coverage, or occupation.

**Table 1:** Demographic characteristics of participants, and correlations with CSQ-8 scores

		**Cebuano CSQ-8 (n = 100)**				**Waray CSQ-8 (n = 106)**			
		**n**	**%**	**r**	**p**	**n**^**†**^	**%**	**r**	**p**
Age	Mean (SD)	30.5	(6.7)	0.07^¶^	0.49	31.6	(5.3)	0.05^¶^	0.64
Number of birth order before the last delivery	Mean (SD)	2.9	(2.4)	0.06^¶^	0.54	3.2	(2.0)	0.03^¶^	0.82
*PhilHealth* coverage	Yes	46	48.4	0.45^§^	0.45	35	55.6	0.11^§^	0.40
	No	49	51.6			28	44.4		
Occupation	Housewife	90	90.9	0.68^§^	0.68				
	Others	9	9.1						

n: number of participants, r: correlation coefficient, p: p-value, ^¶^: Spearman’s rank-order correlation, ^§^: point-biserial correlation, ^†^: total numbers of participants vary due to missing data. For Waray participants, we could not find characteristics of all respondents from the medical records, because in some cases the information given in the interview did not match that written in the medical record (maiden name or married name).

Table 2 shows internal-consistency reliability of the Cebuano CSQ-8. Cronbach’s alpha was 0.85 for the last delivery and 0.86 for the 2nd-to-last delivery. The corrected item-total correlations show that each item in the Cebuano CSQ-8 was consistent with the other items taken together. For each item, Cronbach’s alpha if that item was deleted was no greater than Cronbach’s alpha for the whole scale (0.85 and 0.86 for the last delivery and for the 2^nd^-to-last delivery, respectively).

**Table 2:** Internal-consistency reliability of the Cebuano CSQ-8 (n = 100)

	**The last baby delivery**		**The 2**^**nd**^**-to-last delivery**	
	**Cronbach’s alpha = 0.85**		**Cronbach’s alpha = 0.86**	
**Items (Abbreviated)**	**Corrected item-total correlation**	**Cronbach’s alpha if item was deleted**	**Corrected item-total correlation**	**Cronbach’s alpha if item was deleted**
1. Quality of service	0.54	0.84	0.59	0.85
2. Kind of service	0.61	0.83	0.57	0.85
3. Met need	0.63	0.83	0.61	0.84
4. Recommend to a friend	0.58	0.84	0.68	0.84
5. Amount of help	0.68	0.82	0.66	0.84
6. Deal with problems	0.53	0.84	0.60	0.85
7. Overall satisfaction	0.67	0.83	0.65	0.84
8. Come back	0.52	0.84	0.64	0.85
Suitability for factor analysis				
	Kaiser-Meyer-Olkin value = 0.82		Kaiser-Meyer-Olkin value = 0.85	
	Bartlett’s Test of Sphericity: *p* < 0.001		Bartlett’s Test of Sphericity: *p* < 0.001	

**Figure 2:**
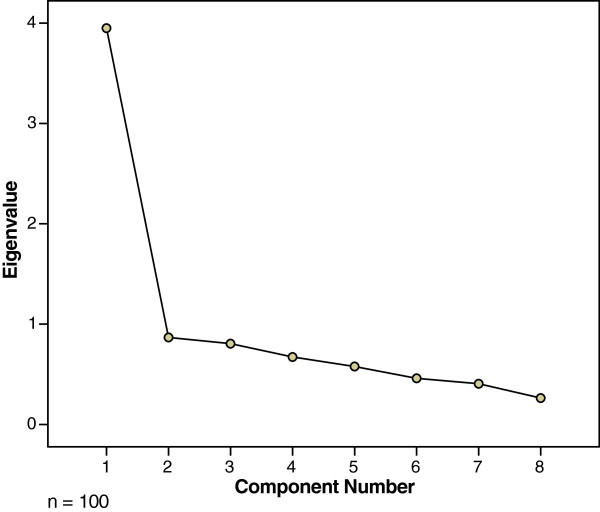
Scree plot of the Cebuano CSQ-8 for the last delivery.

Table 3 shows the component matrix for data regarding the last delivery, for the Cebuano CSQ-8. The Kaiser-Meyer-Olkin value was 0.82, and the result of Bartlett’s Test of Spericity was statistically significant (p < 0.001). There was one component with an eigenvalue exceeding 1, and that one component explained 49.4% of the variance (eigenvalue = 3.95). Figure 2 shows the scree plot, for data regarding the last delivery for the Cebuano CSQ-8. In the scree plot there was a clear break after the first component. In summary, results of the exploratory principal-components analysis suggest that the Cebuano CSQ-8 is unidimensional.

**Table 3:** Component matrix for the Cebuano CSQ-8 for the last baby delivery (n = 100)

**Items**	**Component**							
**(Abbreviated)**	**1**	**2**	**3**	**4**	**5**	**6**	**7**	**8**
1. Quality of service	0.650	-0.174	0.453	-0.520	0.069	-0.031	0.210	-0.147
2. Kind of service	0.718	0.100	-0.398	-0.424	0.186	0.123	-0.141	0.258
3. Met needs	0.738	-0.111	-0.344	0.137	-0.339	0.041	0.433	0.045
4. Recommend to a friend	0.688	0.545	-0.194	0.145	0.300	0.007	0.049	-0.280
5. Amount of help	0.780	-0.107	-0.072	-0.045	-0.439	-0.007	-0.381	-0.186
6. Deal with problems	0.639	-0.518	0.094	0.323	0.295	0.346	-0.062	-0.016
7. Overall satisfaction	0.766	-0.162	0.068	0.205	0.161	-0.545	-0.051	0.121
8. Come back	0.624	0.460	0.517	0.183	-0.166	0.159	-0.021	0.213

For the Waray version, Table 4 shows that Cronbach’s alpha was 0.84 for the last delivery and 0.87 for the 2nd-to-last delivery. The corrected item-total correlations show that each item in the Waray CSQ-8 was consistent with the other items taken together. For each item, Cronbach’s alpha if that item was deleted was no greater than Cronbach’s alpha for the whole scale (0.84 and 0.87 for the last delivery and for the 2^nd^-to-last delivery, respectively).

**Table 4:** Internal-consistency reliability of the Waray CSQ-8 (n = 106)

	**The last baby delivery**		**The 2**^**nd**^**-to-last delivery**	
	**Cronbach’s alpha = 0.84**		**Cronbach’s alpha = 0.87**	
**Items (Abbreviated)**	**Corrected item-total correlation**	**Cronbach’s alpha if item was deleted**	**Corrected item-total correlation**	**Cronbach’s alpha if item was deleted**
1. Quality of service	0.62	0.82	0.60	0.85
2. Kind of service	0.46	0.84	0.52	0.86
3. Met need	0.64	0.81	0.61	0.85
4. Recommend to a friend	0.58	0.82	0.67	0.85
5. Amount of help	0.63	0.82	0.73	0.84
6. Deal with problems	0.62	0.82	0.59	0.86
7. Overall satisfaction	0.58	0.83	0.58	0.86
8. Come back	0.53	0.83	0.68	0.84
Suitability of factor analysis				
	Kaiser-Meyer-Olkin value = 0.85		Kaiser-Meyer-Olkin value = 0.86	
	Bartlett’s Test of Sphericity: *p* < 0.001		Bartlett’s Test of Sphericity: *p* < 0.001	

**Figure 3:**
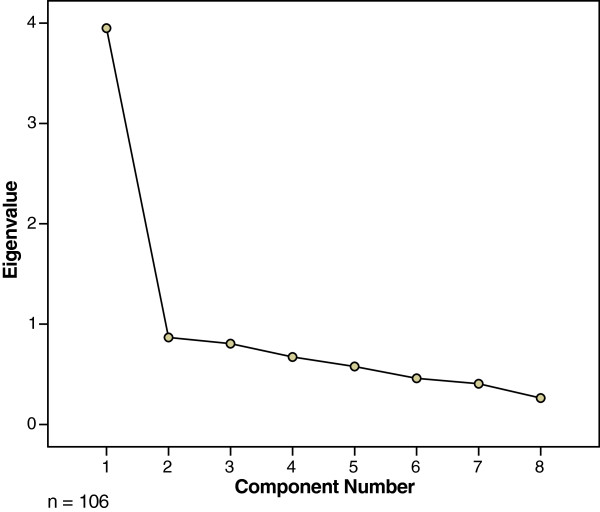
Scree plot of the Waray CSQ-8 for the last delivery.

Table 5 shows the component matrix for data regarding the last delivery, for the Waray CSQ-8. The Kaiser-Meyer-Olkin value was 0.87, and the result of Bartlett’s Test of Spericity was statistically significant (p < 0.001). There was one component with an eigenvalue exceeding 1, and that one component explained 48.7% of the variance (eigenvalue = 3.89). Figure 3 shows the scree plot, for data regarding the last delivery for the Waray CSQ-8. In the scree plot there was a clear break after the first component. In summary, results of the exploratory principal-components analysis suggest that the Waray CSQ-8 is unidimensional.

**Table 5:** Component matrix for the Waray CSQ-8 for the last baby delivery (n = 106)

**Items**	**Component**							
**(Abbreviated)**	**1**	**2**	**3**	**4**	**5**	**6**	**7**	**8**
1. Quality of service	0.739	0.138	-0.124	-0.338	0.417	-0.303	0.128	-0.149
2. Kind of service	0.533	0.291	0.775	-0.081	-0.017	0.132	0.071	-0.039
3. Met needs	0.756	0.204	-0.299	0.050	-0.264	0.181	0.438	0.007
4. Recommend to a friend	0.714	-0.556	-0.003	-0.025	-0.128	0.153	-0.123	-0.355
5. Amount of help	0.728	0.126	0.052	0.416	-0.252	-0.449	-0.119	-0.004
6. Deal with problems	0.732	0.126	-0.138	-0.493	-0.229	0.050	-0.298	0.205
7. Overall satisfaction	0.696	0.301	-0.176	0.384	0.325	0.301	-0.223	0.022
8. Come back	0.667	-0.612	0.139	0.096	0.175	-0.018	0.125	0.325

Table 6 shows comparisons of CSQ-8 scores based on the place of the 2^nd^-to-last delivery. Statistically significant differences were detected in the CSQ-8 scores for Cebuano (p < 0.001, rho = 0.51) and also for Waray (p < 0.001, rho = 0.55). However, regarding the place of the last delivery, the satisfaction scores did not differ significantly: Cebuano (p = 0.72, rho = 0.04) and Waray (p = 0.20, rho = 0.13).

**Table 6:** Comparison of CSQ-8 satisfaction scores between home delivery and facility delivery (the place of 2nd-to-last delivery)

	**Place of delivery**	**The 2nd-to-last delivery**						**The last delivery**					
		**n**	**Median of CSQ-8 scores**	**Mean rank**	**z**	**Asymp. Sig. (2-tailed)**	**Effect size (rho)**	**n**	**Median of CSQ-8 scores**	**Mean rank**	**z**	**Asymp. Sig. (2-tailed)**	**Effect size (rho)**
Cebuano	Home	51	20.0	36.1	-5.08	<0.001	0.51	51	27.0	51.5	-0.35	0.72	0.04
(n = 100)	Hospital	49	27.0	65.5				49	26.0	49.5			
Waray	Home	54	22.0	36.9	-5.69	<0.001	0.55	51	28.0	56.4	-1.30	0.20	0.13
(n = 106)	Hospital	52	28.0	70.8				53	27.0	48.8			

## Discussion

Both the Cebuano and Waray versions of the CSQ-8 have good internal-consistency reliability and both are unidimensional in general satisfaction with childbirth-related care. In both Cebuano and Waray, the internal-consistency reliability of the CSQ-8 was greater than the commonly recommended minimum value for Cronbach’s alpha (0.70).

The results of the factor analyses were generally consistent with the English CSQ-8 in a mental-health setting. The Kaiser-Meyer-Olkin values exceeded the recommended value of 0.6, and the results of Bartlett’s Test of Sphericity were statistically significant, which indicate that factor analysis was appropriate. There was one component with an eigenvalue exceeding 1. Using Catell’s scree test, we decided to retain one component. Similar results were reported with an addict population in Holland [34]. The first component accounted for 65.5% of the total variance, and its eigenvalue was 5.24.

Scores on the Cebuano and Waray versions of the CSQ-8 did not reflect socio-demographic characteristics as clearly as the English CSQ-8 did in a mental-health setting. Overall, the statistical characteristics were similar to those found in a mental-health setting [21].

The construct-validation test was the comparison of the scores for the 2nd-to-last delivery with the scores for the last delivery. As we had hypothesized, the scores for the 2nd-to-last delivery were higher for mothers who had both their second-to-last and their last delivery in a hospital than it was for those who had their 2nd-to-last delivery at home and their last delivery in a hospital. Taken together, these findings indicate that the CSQ-8 can be used to obtain reliable and valid information on satisfaction with childbirth-related services in these areas of the Philippines.

These findings may be encouraging to researchers in other countries that are, like the Philippines, multilingual and multicultural. This is because the findings show how it is possible to simultaneously develop multiple versions of the same instrument, to field-test them for reliability, and to conduct validation tests based on explicit hypotheses. Furthermore, if the CSQ-8 is used to measure satisfaction with childbirth-related services in other countries, then it may be possible to use the resulting scores to compare the needs for services, and the effectiveness of services, between healthcare systems.

As further extensions of the validation testing of the CSQ-8, it might be useful to obtain data from mothers whose 2nd-to-last baby and last baby were both delivered at home. It stands to reason that mothers who were satisfied with the care they had received at home when their 2nd-to-last baby was delivered would not go to a hospital for their next delivery. Likewise, the satisfaction of mothers who self-selected to deliver at a hospital might be inherently high. Also, some mothers might have difficulty expressing dissatisfaction with the services of hospitals while they were still in the post-delivery room, which would result in overestimated satisfaction of care at the hospitals. We conducted the interviews in the post-delivery room because, if there was nothing wrong, the mothers returned home directly from the post-delivery room after the long hours they had spent in the post-delivery room. However, we did not ask the mothers about the name of the hospital where the 2nd-to-last baby had been delivered, and we had no access to mothers whose 2nd-to-last baby and last baby had both been born at home. Data from mothers whose 2nd-to last baby was delivered at a hospital and whose was last baby was delivered at home might also be useful.

One limitation of this study is that we prepared versions in only two Filipino languages. Therefore, participants in this study might not represent all potential mothers in the Philippines. However, those two languages are two of the six major languages in the Philippines. Moreover, those two languages are the major languages in Biliran Province, which recorded the worst MMR in 2005, and Waray is the major language in Eastern Samar, which recorded the worst MMR among provinces in 2009 [35]. Cebuano is widely spoken in Surigao del Sur, which recorded the worst MMR among all provinces in 2008 [36]. The scales we developed should therefore contribute to childbirth-related care in the area of the Philippines where the needs are greatest.

## Conclusion

In conclusion, scores on the CSQ-8 can be used as indices of general satisfaction with childbirth-related services in clinical settings. The CSQ-8 can be used to assess mothers’ satisfaction, so that mothers’ opinions can be taken into account in efforts to improve childbirth-related services, which could increase the proportion of deliveries in medical facilities and thus reduce maternal mortality.

That conclusion applies to scores on the CSQ-8 in two of the six major languages in the Philippines. As the CSQ-8 measures general satisfaction and general satisfaction is less affected by demographics, this study may be a model of a convenient way to develop versions of the CSQ-8 in more than one language. In multilingual countries such as the Philippines, scores on versions of the CSQ-8 in different languages could help policymakers provide better childbirth-related care.

## Competing interests

The authors declare that they have no competing interests.

## Authors’ contributions

CM conceived the study, oversaw the data collection, performed the data analysis, and drafted the manuscript. CM and JG contributed to conceptualizing the study design and analysis methods, interpreting the results, and writing and revising the manuscript. MJ participated in the interpretation of the results and discussions for the manuscript writing and finalization. LTA, ELD, HCJ, and EMG supported the data collection and the coordination of the study. All authors read and approved the final manuscript.

## Pre-publication history

The pre-publication history for this paper can be accessed here:

http://www.biomedcentral.com/1471-2393/13/235/prepub

## Supplementary Material

Additional file 1: Appendix 1 The Client Satisfaction Questionnaire (CSQ-8) for childbirth-related care. Click here for file
