# Supplementary material for: Reliability tests and validation tests of the client satisfaction questionnaire (CSQ-8) as an index of satisfaction with childbirth-related care among Filipino women
Source: BMC Pregnancy Childbirth. 2013 Dec 17;13:235. doi: 10.1186/1471-2393-13-235 (PMC3878559; doi:10.1186/1471-2393-13-235)
Supplement: Additional file 1: Appendix 1 — The Client Satisfaction Questionnaire (CSQ-8) for childbirth-related care. [file 1471-2393-13-235-S1.pdf]

Appendix 1 The Client Satisfaction Questionnaire (CSQ-8) for childbirth-related care

1. How would you rate the quality of service you received?

4 Excellent	3 Good	2 Fair (or moderate)	1 Poor

2. Did you get the kind of service you wanted?

1 No, definitely not	2 No, not really	3 Yes, generally	4 Yes, definitely

3. To what extent has the service met your needs?

4 Almost all of my needs have been met	3 Most of my needs have been met	2 Only a few of my needs have been met	1 None of my needs have been met

4. If a friend were pregnant, would you recommend the service to her?

1 No, definitely not	2 No, I don't think so	3 Yes, I think so	4 Yes, definitely

5. How satisfied are you with the amount of help you received?

1 Quite dissatisfied	2 Indifferent or mildly dissatisfied	3 Mostly satisfied	4 Very satisfied

6. Hove the services you received helped you to deal more effectively with your delivery?

4 Yes, they helped a great deal	3 Yes, they helped somewhat	2 No, they really didn't help	1 No, they seemed to make things worse

7. In an overall, general sense, how satisfied are you with the service you received?

4 Very satisfied	3 Mostly satisfied	2 Indifferent or mildly dissatisfied	1 Quite dissatisfied

8. If you were to seek help again, would you come back to the service?

1 No, definitely not	2 No, I don't think so	3 Yes, I think so	4 Yes, definitely
